# Efficacy and safety of Daoyin and massage for lumbar disc herniation: A protocol for overview of systematic reviews

**DOI:** 10.1097/MD.0000000000028775

**Published:** 2022-02-04

**Authors:** Mingpeng Shi, Xianshuai Zhang, Siyi Wang, Shaojun Li, Changwei Zhao, Zhenhua Li, Jianan Li

**Affiliations:** a Changchun University of Chinese Medicine, China; b Affiliated Hospital of Changchun University of Chinese Medicine, Changchun, Jilin, China; c Tianjin Hospital, Tianjin, China.

**Keywords:** Daoyin, lumbar disc herniation, massage, meta-analysis, overview, systematic review, traditional Chinese medicine

## Abstract

**Background:**

Lumbar disc herniation (LDH) is a common disease, which can cause low back pain, sciatica, and even disability. The treatment of LDH is a global challenge. Conservative therapy with non-drugs is considered to be the first choice for patients with LDH. In recent years, an increasing number of systematic reviews and meta analyses on Daoyin and massage interventions in lumbar disc herniation have been implemented. However, the evidence quality and methodological quality of these systematic reviews/meta analyses are unknown and need to be systematically evaluated. This overview aims to systematically summarize and critically appraise the current evidence on Daoyin and massage for LDH.

**Methods:**

Eight electronic data will be retrieved, including China National Knowledge Infrastructure (CNKI), Wanfang database (WF), China Biomedical database (CBM), Chinese Scientific Journals Database (VIP), PubMed, Cochrane Library, Web of Science (WOS), and EMBASE from their inception to March 1, 2022. The reporting quality, methodological quality, risk of bias, quality of evidence will be assessed by using The Preferred Reporting Items for Systematic Reviews and Meta-analyses 2020 (PRISMA 2020), the Assessment of Multiple Systematic Reviews 2 (AMSTAR-2), the Risk of Bias in Systematic Review (ROBIS), and the Grading of Recommendations Assessment, Development, and Evaluation (GRADE). Two independent researchers conducted literature screening, data extraction, and quality evaluation process. In addition, we will establish an overlap matrix and calculate the corrected covered area to evaluate the impact of overlapping areas on conclusions.

**Results:**

The results will be published in a peer-reviewed journal.

**Conclusion:**

This overview will provide comprehensive evidence of Daoyin and massage for treating lumbar disc herniation.

**Systematic review registration:**

INPLASY202210019.

## 1 Introduction

Lumbar disc herniation (LDH) is the most common cause of low back pain and sciatica.^[1]^ Approximately 70% of people experience low back pain at some point in their life, which has a great impact on people's health and quality of life.^[2]^ A statistical study showed that low back pain has become the main cause of disability all over the world, greatly increasing the economic burden of global rehabilitation needs.^[3,4]^ At present, the surgical treatment of LDH is widely controversial.^[5,6]^ Noninvasive therapies such as spinal massage and functional exercise have become the recommended therapies for LDH in middle-low income countries, which may be the global development trend in the future.^[7]^ For conservative treatment, nonpharmacologic treatment is considered to be the first choice for patients with LDH.^[8]^

As we know, aerobic exercise and resistance training are general exercise strategies to improve low back pain, so the Pilates and McKenzie exercise methods are recommended as effective interventions.^[9,10]^ The Daoyin is a self exercise method based on the theory of traditional Chinese medicine and can improve the disease condition by regulating breathing and exercise.^[11]^ Traditional Chinese medicine (TCM) functional exercises such as Taijiquan, Baduanjin, and Liuzi Jue all belong to the category of Daoyin. Studies have shown that Daoyin can reduce the risk factors of cardiovascular disease, alleviate COPD and improve idiopathic pulmonary fibrosis.^[12–14]^ In China, Daoyin has been widely used in the treatment of lumbar disc herniation as an effective complementary therapy.^[15]^

Although massage has been considered as an effective complementary therapy for LDH,^[8,16]^ the methodological and reporting quality of relevant meta-analysis is still not optimistic, which is difficult to provide high-quality reference evidence for clinicians. A recent case report showed that vigorous massage therapy for a patient with LDH led to cauda equina syndrome, which may make it difficult for health policymakers to judge its safety.^[17]^ Therefore, the evidence and methodological quality of existing systematic reviews (SRs)/meta analyses (MAs) need to be evaluated to draw reliable and comprehensive conclusions.

Daoyin and massage are complementary and alternative therapies based on traditional Chinese medicine theory, and their efficacy in LDH is gradually being confirmed. However, with the significant increase of relevant SRs/MAs in recent years, the methodology and reporting quality of these studies is concerning. Moreover, the evidence quality of these studies is unclear, which is easy to mislead stakeholders when inconsistent conclusions appear. The overview of systematic reviews can integrate SRs/MAs in a given domain to comprehensively evaluate the quality of evidence and literature, thereby providing help for health decision-making.^[18]^ Therefore, this study aims to critically evaluate bias and overlap of the evidence, identify the research gaps, and summarize the best evidence on efficacy and safety of Daoyin and massage for LDH.

## 2 Methods

### 2.1 Protocol and registration

The protocol is registered in the INPLASY (Registration number INPLASY202210019; https://inplasy.com/inplasy-2022-1-0019/). This overview will be performed according to the Preferred Reporting Items for Systematic Reviews and Meta-analyses Protocols (PRISMA-P) when pertinent.^[19]^

### 2.2 Eligibility criteria

The PICOS principle will be adopted in the selection of literature, including participants, intervention, comparison, outcome, and study type.

#### 2.2.1 Type of studies

All published systematic reviews/meta-analyses will be included with no language restrictions, but systematic reviews that only conduct qualitative analysis will be excluded. Meanwhile, the protocols, animal experiments, and other irrelevant literatures will also be excluded.

#### 2.2.2 Type of participants

Patients who are diagnosed with lumbar disc herniation according to standard diagnostic criteria.

#### 2.2.3 Type of interventions

- Massage with or without other therapies
- Daoyin with or without other therapies.

#### 2.2.4 Type of comparisons

Acupuncture, traction, physiotherapy, oral drugs, or other therapies will be included. Placebo or no treatment will also be considered.

#### 2.2.5 Type of outcomes

The primary outcomes will include total effective rate, Oswestry Disability Index scores, and Visual Analogue Scale scores.

The Secondary outcomes will include cure rate, recurrence rate, Japanese Orthopaedic Association scores, SF-36 scale, and adverse events.

### 2.3 Exclusion criteria

- Duplicate publications;
- Non-RCTs included in MAs/SRs;
- Only qualitative analysis was performed in MAs/SRs;
- Overviews, meetings, protocols, meta-analyses of animal experiments.
- Non-full text.

### 2.4 Search strategy

Eight electronic databases will be used to retrieve literature, including China National Knowledge Infrastructure (CNKI), Wanfang database (WF), China Biomedical database (CBM), Chinese Scientific Journals Database (VIP), PubMed, Cochrane Library, Web of Science (WOS), and EMBASE from their inception to March 1, 2022. Literature search will be conducted by 2 reviewers (MS and XZ). The search terms include “Daoyin”, “Taiji”, “Baduanjin”, “Yanfei”, “Wuqinxi”, “Yijinjing”, “Massage”, “lumbar disc herniation”, “meta-analysis” and “systematic review”. Taking PubMed database as an example, the search terms are shown in Table 1.

**Table 1:** Search strategy used in PubMed database.

Query	Search term
#1	“lumbar disc herniation”[Title/Abstract] OR “LDH”[Title/Abstract] OR “lumbar disc protrusion”[Title/Abstract] OR “lumbar disk herniation”[Title/Abstract] OR “lumbar herniated disk”[Title/Abstract] OR “lumbar intervertebral disc herniation”[Title/Abstract] OR “lumbar intervertebral disc prolapse”[Title/Abstract] OR “LIDP”[Title/Abstract] OR “prolapse of lumbar intervertebral disc”[Title/Abstract]
#2	“Traditional Chinese exercise”[Title/Abstract] OR “Daoyin”[Title/Abstract] OR “guidance”[Title/Abstract] OR “Qigong”[MeSH] OR “Qi Gong”[Title/Abstract] OR “Ch’i Kung”[Title/Abstract] OR “Tai ji”[MeSH] OR “Tai Chi”[Title/Abstract] OR “Chi, Tai”[Title/Abstract] OR “Tai Ji Quan”[Title/Abstract] OR “Taijiquan”[Title/Abstract] OR “Five animal Exercise”[Title/Abstract] OR “Wu Qin Xi”[Title/Abstract] OR “Wuqinxi”[Title/Abstract] OR “Ba Duan Jin”[Title/Abstract] OR “Baduanjin”[Title/Abstract] OR classics of tendon changing”[Title/Abstract] OR “Yi Jin Jing”[Title/Abstract] OR “Yijinjing”[Title/Abstract] OR “Liuzi Jue”[Title/Abstract] OR “Six words”[Title/Abstract] OR “Massage”[MeSH] OR “Therapy, Massage”[Title/Abstract] OR “Zone Therapy”[Title/Abstract] OR “Zone Therapies”[Title/Abstract] OR “Therapies, Zone”[Title/Abstract] OR “Therapy, Zone”[Title/Abstract] OR “Tuina”[Title/Abstract] OR “Acupressure”[Title/Abstract] OR “Bone setting”[Title/Abstract]
#3	“Systematic Review” [Publication Type] OR “Systematic Reviews as topic”[MeSH] OR “Network Meta-Analysis”[MeSH] OR “Meta-analysis”[Publication Type] OR “Meta-analysis as topic”[MeSH] OR “Systematic review”[Title/Abstract] OR “Meta-analysis”[Title/Abstract]
#4	#1 AND #2 AND #3

### 2.5 Selection of studies and data extraction process

**Figure 1:**
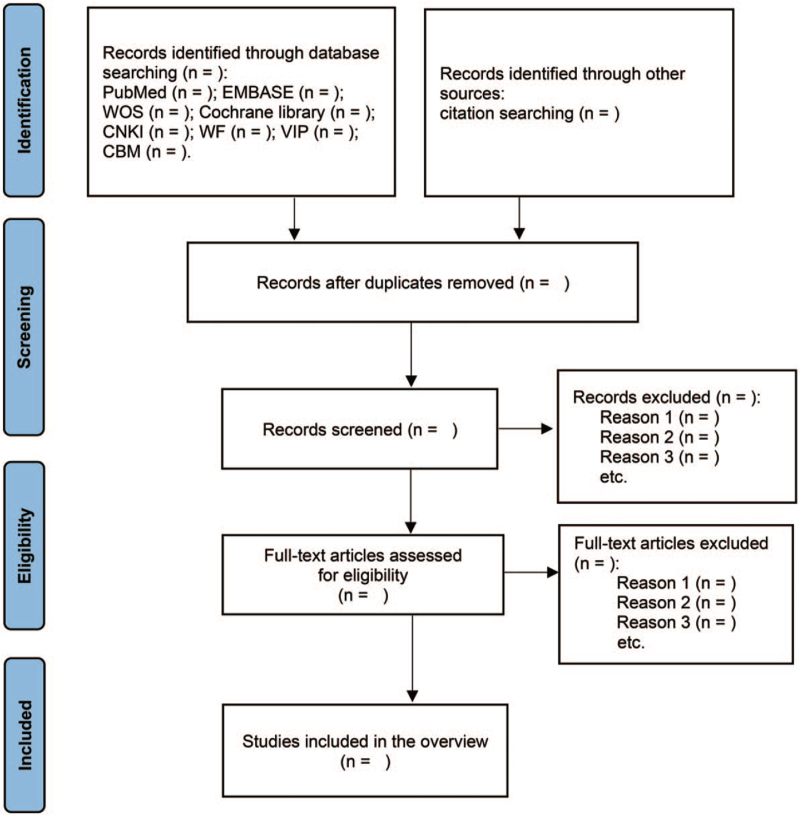
Flow chart of literature selection.

According to the pre-set inclusion and exclusion criteria, 2 evaluators (MS and XZ) will screen the preliminarily searched literature by reading the title, abstract or full text. Noteexpress 3.4 (Beijing Aegean hailezhi Technology Co., Ltd., Beijing, China) will be used for literature screening and management. If there is a disagreement between the reviewers during screening, the arbitrator (SL) will decide whether to include the controversial literature. We will search for references from included literature as a supplementary retrieval method. The literature screening process and results will be presented in Figure 1. If possible, we will provide a list of literature excluded by reading the full text.

Two reviewers (MS and XZ) will independently extract data using Excel 2019, and the extracted data include author, year, language, publication type, type of primary study, number of included studies and cases, characteristics of interventions and controls, tools and results for assessing the quality of studies, outcome indicators, main results (meta-analysis) and key conclusions. The results of data extraction will be cross-checked by the 2 reviewers to verify their accuracy. In case of disagreement, the third reviewer (SL) will participate in the discussion and make the final decision.

Adverse events will be extracted from all the included studies to draw a table, including whether to report, the number of occurrences, the results of the meta-analysis, the type and severity of the adverse event, and how to deal with it.

### 2.6 Assessment of report and methodological quality

The PRISMA 2020 statement that contains a total of 27 items (42 sub-items) will be used to assess the report quality of included SRs.^[20,21]^ For each item, the complete report will be recorded as “1” point, the unclear or partial report will be recorded as “0.5” point, and the no report will be recorded as “0” point, with a full score of “42” points. The final total score of each article will be used to compare the reporting quality. Referring to the PRISMA 2020 statement, 2 senior reviewers (JL and CZ) will evaluate the literature independently, and the controversial literature will be arbitrated by the third evaluator (ZL).

The AMSTAR-2 will be used to evaluate the quality of methodology of included SRs.^[22]^ It contains 16 items (evaluated with “yes”, “partial yes”, and “no”), and the final evaluation results will show the quality of methodology with “high”, “moderate”’, “low”, or “very low”. Two reviewers (JL and CZ) will evaluate the quality of methodology in included studies using the AMSTAR-2 tool, and in case of disagreement, it will be arbitrated by a third party (ZL).

### 2.7 Assessment of bisk of bias

The ROBIS tool will be used to evaluate the risk of bias in the included reviews, which consists of 3 evaluation stages and signaling questions.^[23]^ The final bias risk assessment results are “high”, “low” or “uncertain”. Considering that we evaluate the risk of bias of included SRs rather than consistency between the problem to be solved and the actual problem, we only use the second and third stages of ROBIS. Similarly, we will adopt the method of independent evaluation by 2 evaluators. If the evaluation results of the 2 evaluators (JL and CZ) are inconsistent, the third evaluator (ZL) will arbitrate.

### 2.8 Assessment of quality of evidence

Two reviewers (JL and CZ) will assess the quality of evidence by using the grading of recommendations, assessment, development, and evaluation (GRADE). The GRADE system includes 5 degradation areas: risk of bias, inconsistency, indirectness, imprecision, and publication bias. If the relevant upgrade conditions are met, rating up the quality of evidence will be considered. The evaluators will classify the evaluation results as “high”, “medium”, “low” or “very low”. In case of disagreement, it will be decided by the third evaluator (ZL).

### 2.9 Assessment of overlapping areas

In the included reviews, the primary study may be included in 2 or more systematic reviews. These repeatedly included studies may exaggerate the efficacy and lead to similar conclusions in meta-analyses.^[24]^ Therefore, we will list an overlap matrix that contains only index publications as a supplemented appendix. In addition, we will use the corrected covered area (CCA) methods to assess the degree of overlapping areas.^[25]^

If the CCA is “0∼5” indicates slight overlap, “6∼10” indicates moderate overlap, “11∼15” indicates high overlap, and “CCA≥15” indicates very high overlap. Finally, we will report the CCA of Daoiyin and massage respectively.

### 2.10 Data synthesis

We will summarize all the results included in the SRs/MAs through a narrative description, and analyze the reliability of the conclusions based on the quality assessment results. In addition, we will provide comprehensive conclusions on massage and Daoyin for treating LDH, respectively.

If feasible, we will conduct exploratory subgroup analyses for each outcome according to the frequency or type of Daoyin. Before the quantitative synthesis, we will eliminate the duplicate primary studies of included reviews, which means that only the index publications will be retained. If necessary, a meta-analysis will be performed using RevMan 5.3 (The Cochrane Collaboration 2014). For continuous outcome, the results will be expressed as mean difference (MD) with 95% confidence interval (CI), and for dichotomous data, odds ratio (OR) with 95% CIs will be used. The random effects model and fixed effects model will be selected according to the level of heterogeneity described by the *I*^2^ value. When *I*^2^≥50%, the random effects model will be performed, otherwise the fixed effects model will be performed. If available, we will conduct sensitivity analysis by removing 1 study at a time to verify the stability of these results.

## 3 Discussion

This protocol aims to critically evaluate the efficacy and safety of Daoyin and massage for treating LDH, and we expect that the final comprehensive conclusions will serve health decision-making and stakeholders. The CCA will be calculated to assess the degree of overlap of primary studies included in SRs, which is helpful to judge the impact of overlap on research conclusions. In addition, we will highlight the defects in the included SRs/MAs, which can help researchers improve the methodology and reporting quality of relevant studies.

Few limitations should be considered in this study. First, most studies on Daoyin and massage for treating LDH are implemented in China, and the conclusion may be affected by regional differences. Second, only published studies will be included, which could introduce reporting bias into our study. Despite these limitations, this study can still provide reliable conclusions on the efficacy and safety of Daoyin and massage for LDH.

## Author contributions

**Conceptualization:** Zhenhua Li, Mingpeng Shi, Jianan Li.

**Data curation:** Mingpeng Shi, Xianshuai Zhang, Shaojun Li, Jianan Li, Zhenhua Li, Changwei Zhao.

**Formal analysis:** Siyi Wang.

**Methodology:** Zhenhua Li, Jianan Li.

**Software:** Siyi Wang.

**Supervision:** Zhenhua Li.

**Writing – original draft:** Mingpeng Shi, Xianshuai Zhang.

**Writing – review & editing:** Zhenhua Li.
